# Catastrophic healthcare expenditure and coping strategies among patients attending cancer treatment services in Addis Ababa, Ethiopia

**DOI:** 10.1186/s12889-020-09137-y

**Published:** 2020-06-22

**Authors:** Gebremicheal Gebreslassie Kasahun, Gebremedhin Beedemariam Gebretekle, Yohannes Hailemichael, Aynalem Abraha Woldemariam, Teferi Gedif Fenta

**Affiliations:** 1 Department of Pharmacy, College of Health Sciences, Aksum University, Aksum, Tigray Ethiopia; 2 Department of Pharmaceutics and Social Pharmacy, School of Pharmacy, College of Health Sciences, Addis Ababa University, Addis Ababa, Ethiopia; 3 School of Public Health, College of Health Sciences, Addis Ababa University, Addis Ababa, Ethiopia; 4 School of Medicine, College of Health Sciences, Addis Ababa University, Addis Ababa, Ethiopia

**Keywords:** Catastrophic health expenditure, Coping strategy, Out of pocket expenditure, Cancer

## Abstract

**Background:**

With the rapid increase in magnitude and mortality of cancer, which is costly disease to manage, several patients particularly in developing countries are facing a huge financial burden. The study aimed to examine the incidence of catastrophic health expenditure (CHE), identify associated factors and coping strategies among patients attending cancer treatment services in Addis Ababa, Ethiopia.

**Methods:**

A hospital-based cross-sectional survey of patients with cancer was conducted in public and private hospitals between January and March 2018. Data was collected using a structured questionnaire. All direct medical and nonmedical expenditures were measured and reported as expenditure (US$) per patient (1US$ equivalent to 23.41 Ethiopian Birr). The CHE was estimated using a threshold of 10% of annual household income.

**Results:**

A total of 352 (response rate of 87.1%) participants were interviewed. Majority (73.3%) of the respondents were females; most (94%) from public hospitals and their mean (±SD) age was 48 ± 13.2 years. Vast majority (74.4%) of patients experienced CHE with mean overall expenditure of $2366 per patient (median: $1708). Medical expenditure shared the highest overall expenditure (83.6%) with mean medical and nonmedical costs of $1978 (median: $1394) and $388 (median: $222), respectively. Patients who took greater than six cycles of chemotherapy (AOR: 3.64; 95% CI: 1.11–11.92), and age (AOR: 1.03; 95% CI: 1.01–1.06) were significantly associated with CHE. Household saving (85.5%) followed by financial support received (43.0%) was the main coping strategy.

**Conclusion:**

A substantial number of patients with cancer were exposed to CHE with a considerable medical expenditure. Hence, in addition to the popularization of the already introduced health insurance scheme, other better prepayment or insurance mechanisms should also be considered to ensure financial risk protection and realize universal health coverage for patients with cancer.

## Background

Out of pocket (OOP), healthcare financing leaves households exposed to the risk of unanticipated catastrophic financial expenditures that absorb a large share of the household budget [1]. Catastrophic health expenditure (CHE) is healthcare spending that exceeds some specified critical level of tolerance or threshold from the household total income in a given specified period [2–5]. According to Wagstaff and Van Doorslaer [4], the incidence of CHE is estimated from the fraction of OOP payments, which exceeds a certain threshold, usually 10% of total household annual income, often for one-year interval. It occurs when the available health service is mainly dependent on OOP payment, households have low capacity to pay, and if there is no prepayment [6]. CHE is highly pronounced when the diseased household member is at a productive age, and when the individual is the primary source of household income [1].

Cancer is one of the chronic non-communicable diseases with a high likelihood of imposing CHE [7]. It is a collection of conditions in which cells change, multiply and metastasize out of control in the body. Lung, cervical, prostate, stomach, colorectal, liver, and breast are among the most prevalent cancer types in the world [8]. According to the world health organization (WHO) global cancer report, about 14.1 million new cancer cases and 8.2 million deaths occurred in 2012 globally. The global burden of cancer is projected to continue, particularly in developing countries. It is projected to increase to 19.3 million new cancer cases per year by 2025 worldwide [9]. It imposed the most devastating economic impact in 2010; about 808 million people had incurred catastrophic health spending worldwide [10]. Moreover, due to premature death and disability, about $895 billion of economic impact was debited in 2008 globally without including the direct medical cost, which could further increase the financial lost [11].

Like other countries, especially low-middle-income countries [12], there is a rapid increase in the magnitude and mortality of cancer in Ethiopia. Besides, it is believed that cancer is costly to manage; as a result, several patients are facing a huge financial burden. Intending to share financial risks and improving access to essential services, Ethiopia’s government had developed a strategic plan for establishing and expanding mandatory health insurance [13]. Cancer is among the services covered by the insurance scheme, but its coverage remains very low in pilot phase [14]. As a result, the majority of the population is still dependent on OOP payment for healthcare service. On the other hand, there is limited evidence about the level of CHE and OOP healthcare expenditure for the diagnosis and treatment of cancer in Ethiopia [15, 16]. Therefore, the study aimed to examine the incidence of CHE, identify associated factors and coping strategies among patients attending cancer treatment services in Addis Ababa, Ethiopia.

## Methods

### Study area and study design

Hospital-based cross-sectional study design was employed. The study was conducted in Addis Ababa, the capital city of Ethiopia (the second-most populous country in Africa) and headquarters of African Union. Addis Ababa has 13 public hospitals, 32 private hospitals, and 93 health centers. From ten cancer diagnostic and treatment centers, about 150–200 new cases of cancer are registered monthly. Each month, about 1200 cancer patients visit these health facilities either for follow up or treatment. From the public hospitals, Tikur Anbessa Specialized Hospital (TASH) is the biggest teaching and referral public hospital with the highest number of cases. The study was conducted in TASH and three private hospitals (Hallelujah, Bete-Zata and Leghar General Hospitals) between January and March 2018.

### Study population and sampling procedure

The source population was all cancer patients attending treatment service in Addis Ababa healthcare facilities. Histological and/or pathologically confirmed cancer patients, patients who took at least one of the anticancer therapy options (chemotherapy, radiotherapy or hormonal/others), patients with a regular follow up for the last 1 year preceding the interview date, and with no co-morbidity and were volunteer to participate, constituted the study participants.

The sample size was determined using a single proportion population formula [17], and a total of 404 participants were recruited from one government hospital (TASH) and three private hospitals. TASH was included as it is the sole oncology referral and radiotherapy center in the country and it has also the highest number of cancer patients. Private hospitals were also selected based on their voluntariness to be enrolled and patient load. Participants were recruited using a convenience sampling technique and the number of participants to be drawn from each hospital was decided based on proportion to patient load.

### Data collection and management

A structured questionnaire (S1 Table 1 Data collection instrument) was developed from the WHO SAGE (Study of Global Ageing and Adult Health) study [18] and other relevant literatures [15, 19, 20]. This English version of the questionnaire was translated into Amharic (national language) and back-translated to English to ensure consistency. The questionnaire was pretested before the commencement of the data collection process. Appropriate modification and validation process were made based on the result of the pretest.

The data collection tool was consisted of six parts: (1) socio-demographic characteristics (age. Gender, occupation, marital status, education level and household size); (2) medical information (such as type of cancer, time of first diagnosis, treatment initiated, type of treatments taken and history of visit to a private health facility); (3 and 4) expenditures on the outpatient and inpatient services (including consultation, investigation, medicine and other relevant costs); (5) household essential consumption and income (weekly food and others spending, monthly house rent, cloths, transport and other charges, annual education payment, durable materials e.g., television, phone, furniture, vehicles, ceremonies and others spending, overall household yearly expenditure and income, and patient income if available); and (6) households financial situation outlook (rate of financial burden, coping mechanisms taken and its amount). Besides, patient medical charts were reviewed to collect relevant clinical information, investigations and treatments taken which also helped in estimating patient treatment/diagnostic expenditures.

Data was collected in a face-to-face exit interview and daily supervision was made by the principal investigator to ensure its completeness and consistency.

### Measurements

The magnitude of CHE was estimated using Wagstaff and Van Doorslaer [4] approach. When previous one-year patient households’ OOP expenditure for cancer care exceeded 10% of total annual household income, it was considered catastrophic. The overall, outpatient and inpatient cancer diagnostic and treatment service expenditures of the last 12 months were estimated and presented as average expenditure per patient. Therefore, these households incurred a catastrophic expenditure assumed as catastrophic payment headcounts (H_cat_).

Healthcare service OOP expenditure is a direct payment made to healthcare providers on receiving a service excluding prepayment, reimbursement, and other sources of payment mechanisms [4, 5, 21]. However, households can use different coping strategies to solve their financial burden. Coping mechanisms may include any means of income obtained from household member and other savings including *Eqqub* and *Iddir*, any financial support from relatives, religious organization and other sources which are non-refundable. Income derived from selling household assets like property, livestock, jewellery, and other household items; and any types of borrowing from financial institutions or individuals could be sources of finance to cover any medical and non-medical expenses. These coping mechanisms could be rearranged as; savings, financial support, selling assets, and borrowings [7, 20]. Households had used different coping strategies, which can be classified into two categories: expenditure covered by only the households themselves and by other mechanisms to estimate the CHE level of cancer care.

Households’ subjective rate of self-reported financial burden was assessed by asking respondents to rate their current household economic situation compared to the past; as very good, good, medium/similar, bad and worse. Later, they were reclassified as “manageable” for very good, good and medium/similar and “unmanageable” for bad and worse.

Expenditure was estimated as all costs spent for cancer care before the interview time in the last year of medical service upon possible probing approaches to minimize recall bias. This expenditure includes both medical and non-medical expenses. Medical expenses were all expenditures related to consultation, investigation, medicine, bed and traditional medicine. On the other hand, non-medical cost includes transportation, food and other accommodation expenditures associated with the patient care. Expenses were also categorized as outpatient expenditures (consultation, investigation, medicine, and other spending related to outpatient care), and inpatient expenditures (consultation, investigation, medicine, bed and other expenses associated with inpatient care). There are controversies on estimating cost data, especially when the data have a skewed nature. Some studies reported “mean” as a reasonable choice, although it could be affected by the extreme values [22, 23]. However, others studies preferred to report using the median since it is not affected by skewness, although it only shows the position of the distribution [24]. Hence, we used both mean and median measurements to provide a full picture of the estimation, which might ease health policy decision making and resource allocation.

Household income and expenditure were measured based on respondents’ self-reported daily or monthly income and expenditure. Participants with in-kind income were approached for their type of income and amount. The in-kind income was then changed to monetary terms based on their current value to the local market. Besides, all expenditures were measured, and costs were reported as an expenditure (US$) per patient (1US$ equivalent to 23.41 Ethiopian Birr).

### Covariates

Socio-demographic, clinical and economic variables including type of health facility (private and public), kind of cancer (breast cancer, colorectal cancer, cervical cancer, nasopharyngeal cancer (NPC) and others), cycles of chemotherapy taken (less than or equal to three cycles, four to six cycles, more than six cycles and on other treatment options), history of a visit to a private health facility, gender, marital status,residence (from Addis Ababa and outside of Addis Ababa), the level of education, occupation, income, and expenditure were the variables considered as covariates in the analysis.

### Statistical analysis

Data were coded, entered in to, and analyzed using STATA version 14 (https://www.stata.com/stata14/). Descriptive statistics such as mean, median, standard deviation (SD) and Inter-Quartile Range (IQR) were used to compute socio-demographic, clinical and economic characteristics. Hosmer-Lemeshow test was employed for the goodness of fit test of the logistic regression model. A multivariable logistic regression model was used to assess the relationship between CHE and potential explanatory variables. The significance level was set at 5 and 95% confidence interval (CI).

## Results

### Socio-demographic and clinical information

A total of 404 participants were approached, and 352 study participants were interviewed, with a response rate of 87.1%. The majority (73.3%) of the patients were females, and 285 (81.0%) were married. Most (94%) of the respondents were from public hospitals (Table 1). More than three-fourth (85.2%) of the study participants were in the productive age category, and their mean (±SD) age was 48 (±13.2) years (ranged 19 to 87 years).

**Table 1:** Socio-demographic and economic characteristics among patients attending cancer treatment services in Addis Ababa, Ethiopia 2018

Characteristics	Frequency (N)	Percentage (%)
Type of health facility		
Public	331	94.0
Private	21	6.0
Gender		
Female	258	73.3
Male	94	26.7
Age (mean, ±SD)	48^a^	13.2^b^
Marital status		
Married	285	81.0
Single	34	9.6
Divorced	19	5.4
Widowed	14	4.0
Residence		
Out of Addis Ababa	209	59.4
Addis Ababa	143	40.6
Education		
No formal education^c^	127	36.1
College/certificate and above	122	34.6
Grade 9–12	69	19.6
Grade 1–8	34	9.7
Occupation		
Housewife/Husband	139	39.5
Employed (private/government)	102	29.0
Own private business	49	13.9
Retired	36	10.2
Others^d^	26	7.4
Household economic income quintile		
Lowest	74	21.0
Second	74	21.0
Middle	64	18.2
Fourth	71	20.2
Highest	69	19.6
Household economic expenditure quintile		
Lowest	71	20.2
Second	70	19.9
Middle	72	20.5
Fourth	85	24.1
Highest	54	15.3

^a^: age (Mean),

^b^: age (SD = Standard deviation),

^c^: include illiterates and individuals able to read and write but not attended formal education,

^d^: include individuals who are not employed/stopped to work

More than one-third (36.9%) of the participants were breast cancer patients, followed by cervical cancer 58 (16.5%) and colorectal cancer 46 (13.1%). The majority (89.0%) of patients have taken chemotherapy on their treatment course, and 155 (44.0%) of them were on 4–6 cycles of chemotherapy. All patients have used supportive treatments. The majority (63.4%) of respondents had a history of visit at private health facilities during the course of the disease (Table 2).

**Table 2:** Clinical information of patients attending cancer treatment services in Addis Ababa, Ethiopia 2018

Clinical variables	N	% (95% CI)
Type of cancer		
Breast cancer	130	36.9 (32.0–42.1)
Cervical cancer	58	16.5 (13.0–20.7)
Colorectal cancer	46	13.1 (9.9–17.0)
NPC^a^	13	3.7 (2.2–6.3)
Others^b^	105	29.8 (25.3–35.0)
Type of treatments taken^c^		
Supportive treatment	352	100.0
Chemotherapy	313	89.0 (85.2–91.8)
Surgery	201	57.1 (51.8–62.2)
Radiotherapy	176	50.0 (44.8–55.2)
Hormonal	80	22.7 (18.6–27.4)
Cycle of treatment taken		
4–6 cycles	155	44.0 (38.9–49.3)
1–3 cycles	97	27.6 (23.1–32.5)
> 6 cycles	61	17.3 (13.7–21.7)
On other treatment options	39	11.1 (8.2–14.8)
Private health facility visit history		
Yes	223	63.4 (58.2–68.2)
No	129	36.6 (31.8–41.8)

^a^: *NPC* Nasopharyngeal cancer,

^b^: Other cancer types (including skin, oral, lung, liver, esophageal, bone, Hodgkin lymphoma, non-Hodgkin lymphoma, thyroid and other unspecified tumors, confirmed on histological and/or pathologically exam)

^C^: Frequencies and percentage will not be added up because multiple responses were possible

### Overall, outpatient and inpatient services expenditure

The average overall expenditure per patient in the last one-year was estimated to be $2366 (SD: $4262), median: $1709 (IQR: $1153–2424). The inpatient services accounted for two-thirds of the total expenditure with a mean cost of $1584 (SD: $4002), and a median of $1067 (IQR: $641–1580) per patient. The remaining expenditure was spent on outpatient service, with a mean of $782 (SD: $1468) and a median of $557 (IQR: $256–940).

The overall mean expenditure of patients with colorectal cancer who attended private hospitals, who had a history of visit at private health facilities, and those living out of Addis Ababa was higher than their counterparts (Table 3).

**Table 3:** Mean overall expenditure per patient by different subgroups among patients attending cancer treatment services in Addis Ababa, Ethiopia 2018

Subgroup variables	Mean overall expenditure per patient (SD) ($)
By type of cancer	
Breast cancer	2325 (2776)
Cervical cancer	1308 (722)
Colorectal cancer	2931 (3287)
NPC	1652 (486)
Others	2842 (6751)
By type of health facility	
Private	5341 (10814)
Government	2177 (3410)
By history of visit at private health facility	
Visited	2870 (5256)
Not visited	1494 (822)
Have no history of visit at private health facility by type of cancer	
Breast cancer	1658 (923)
Cervical cancer	1074 (581)
Colorectal cancer	2144 (806)
NPC	–
Others	1446 (653)
Have a history of visit at private health facility by type of cancer	
Breast cancer	2799 (3479)
Cervical cancer	1616 (783)
Colorectal cancer	3150 (3673)
NPC	1652 (486)
Others	3454 (8035)
Catastrophic	2495 (4745)
By their catastrophic level	
Not catastrophic	1990 (2324)
By gender	
Male	2704 (5293)
Female	2243 (3822)
By marital status	
Single	3751 (8255)
Divorced	2598 (1873)
Married	2244 (3736)
Widowed	1174 (761)
By residence	
Addis Ababa	2223 (4310)
Out of Addis Ababa	2464 (4236)
By educational level	
No formal education	1674 (913)
Grade 1–8	1685 (771)
Grade 9–12	2306 (2986)
College/certificate and above	3310 (6716)
By occupational status	
Retired	3229 (8288)
Employed (private/government)	2455 (3024)
Own private business	2396 (2038)
Housewife/Husband	2164 (4488)
Others	1842 (1022)

---: There are no cases of NPC with history of visit at private health facility; *SD* Standard deviation; 1 US$ =23.41 Ethiopian Birr (ETB)

### Medical and non-medical expenditure

The direct mean and median medical expenditures per patient in the last year was estimated to be $1978 (SD: $3554) and $1394 (IQR: $917–1982), respectively. On the other hand, the patients had a mean and median non-medical expenditure of $388 (SD: $993) and $222 (IQR: $122–461), respectively. The medical spending constituted the highest expenditure (83.6%), taking a large share of the overall expenditure. The mean cost on traditional medicines was also estimated to be $7 (SD: $65).

### The magnitude of catastrophic healthcare expenditure

The average headcount adult equivalent income and expenditure was found to be $1821 (median: $1220) and $1111 (median: $949) per year, respectively. The mean annual unadjusted household income and expenditure was estimated to be $4997 (median: $3076) and $3006 (median: $2563) per year, respectively. The incidence of incurring CHE was 74.4% (95%, CI: 69.6–78.7). Patients in the fourth income quintile had faced higher CHE. Regarding the expenditure quintile, patients in the middle quintile had encountered higher catastrophic cancer care expenditure (Table 4).

**Table 4:** Factors associated with CHE and proportion of CHE among subgroups of patients attending cancer treatment services in Addis Ababa, Ethiopia 2018

Variables	Proportion of CHE N, % (95% CI)	COR (95% CI)	AOR (95% CI)
Type of Health Facility			
Private	16, 76.2 (53.3–89.9)	1.11 (0.39–3.11)	1.29 (0.38–4.40)
Public^a^	246, 74.3 (69.3–78.7)	1.00	1.00
Type of cancer			
Breast cancer	94, 72.3 (63.9–79.4)	1.10 (0.55–2.14)	0.72 (0.31–1.66)
Colorectal cancer	32, 69.6 (54.7–81.2)	0.95 (0.41–2.21)	0.68 (0.22–2.10)
NPC	11, 84.6 (53.3–96.3)	2.28 (0.46–11.40)	2.92 (0.41–20.7)
Others	84, 80.0 (71.2–86.6)	1.66 (0.79–3.48)	1.33 (0.52–3.43)
Cervical cancer^a^	41, 70.7 (57.6–81.0)	1.00	1.00
Cycles of chemotherapy taken			
≤ 3 cycles	71, 73.2 (63.4–81.1)	1.21 (0.54–2.74)	1.25 (0.47–3.35)
4–6 cycles	113, 72.9 (65.3–79.3)	1.20 (0.55–2.57)	1.31 (0.52–3.43)
> 6 cycles	51, 83.6 (72.0–91.0)	2.27 (0.87–5.92)	**3.64 (1.11–11.92)**
On other treatment options^a^	27, 69.2 (53.0–81.8)	1.00	1.00
Private health facility visit			
No	97, 75.2 (67.0–82.0)	1.06 (0.65–1.75)	1.19 (0.66–2.13)
Yes^a^	165, 74.0 (67.8–79.3)	1.00	1.00
Age	–	**1.01 (0.99–1.03)**	**1.03 (1.01–1.06)**
Household size	–	0.95 (0.87–1.04)	0.95 (0.85–1.07)
Gender			
Male	72, 76.6 (66.9–84.1)	1.17 (0.67–2.03)	1.01 (0.41–2.46)
Female^a^	190, 73.6 (67.9–78.7)	1.00	1.00
Marital status			
Single	26, 76.5 (59.2–87.9)	1.14 (0.50–2.63)	1.91 (0.60–6.13)
Divorced	14, 73.7 (49.4–88.9)	0.98 (0.34–2.82)	1.20 (0.34–4.20)
Widowed	11, 78.6 (49.2–93.2)	1.29 (0.35–4.74)	1.48 (0.31–6.96)
Married^a^	211, 74.0 (68.6–78.8)	1.00	1.00
Residence			
Out of Addis Ababa	158, 75.6 (69.3–81.0)	1.16 (0.72–1.89)	1.07 (0.61–1.86)
In Addis Ababa^a^	104, 72.7 (64.8–79.4)	1.00	1.00
Level of Education			
No formal education	93, 73.2 (64.8–80.2)	0.93 (0.53–1.64)	0.47 (0.18–1.26)
Grade 1–8	28, 82.4 (65.5–92.0)	1.59 (0.60–4.20)	0.99 (0.30–3.26)
Grade 9–12	50, 72.5 (60.7–81.8)	0.89 (0.46–1.75)	0.59 (0.25–1.42)
College and above^a^	91, 74.6 (66.1–81.6)	1.00	1.00
Occupation			
Own private business	39, 79.6 (65.8–88.7)	1.40 (0.62–3.20)	2.21 (0.79–6.17)
Housewife/Husband	107, 77.0 (69.2–83.3)	1.21 (0.67–2.17)	2.30 (0.87–6.01)
Retired	22, 61.1 (44.5–75.6)	0.56 (0.25–1.26)	0.48 (0.17–1.33)
Others	19, 73.1 (52.8–86.8)	0.98 (0.37–2.58)	1.17 (0.30–4.55)
Employed private/government^a^	75, 73.5 (64.1–81.2)	1.00	1.00
Income quintile			
Lowest	49, 66.2 (54.6–76.1)	1.04 (0.52–2.09)	0.85 (0.18–3.86)
Second	58, 78.4 (67.4–86.4)	1.93 (0.92–4.06)	1.30 (0.38–4.38)
Middle	50, 78.1 (66.2–86.7)	1.91 (0.88–4.12)	1.21 (0.41–3.57)
Fourth	60, 84.5 (74.0–91.3)	2.91 (1.29–6.55)	2.66 (0.97–7.29)
Highest^a^	45, 65.2 (53.2–75.6)	1.00	1.00
Expenditure quintile			
Lowest	48, 67.6 (55.8–77.5)	1.27 (0.58–2.58)	1.49 (0.30–7.34)
Second	51, 72.8 (61.2–82.1)	1.58 (0.74–3.38)	1.52 (0.42–5.50)
Middle	62, 86.1 (75.9–92.4)	3.65 (1.53–8.68)	3.04 (0.91–10.17)
Fourth	67, 78.8 (68.7–86.3)	2.19 (1.02–4.67)	1.49 (0.52–4.33)
Highest^a^	34, 63.0 (49.3–74.8)	1.00	1.00

^a^: reference group; Bold: significant association at 95% CI (COR and AOR); *AOR* Adjusted odds ratio; *COR*:Crude odds ratio

Based on participant’s self-report, it was estimated that 69% (95% CI: 64.0–73.7) of patients’ households faced with an unmanageable financial burden which could lead them to a financial crisis.

### Factors associated with catastrophic health expenditure

The multivariable logistic regression analysis revealed that taking greater than six cycles of chemotherapy (AOR: 3.64; 95% CI: 1.11–11.92), and age (AOR: 1.03; 95% CI: 1.01–1.06) were factors statistically associated with CHE among cancer patients. In this regard, the tendency to encounter CHE of cancer care was associated with older age and an increased number of cycles of chemotherapy (Table 4).

### Financial burden coping strategies of cancer diagnosis and treatment

The majority of households had used their household savings (85.5%) for healthcare payments. However, a considerable percentage (43%) of households have received substantial financial support from their relatives, religious and other non-governmental organizations to cover the financial burden imposed on the household. The remaining expenditure was covered by selling assets and borrowings, as illustrated in Table 5.

**Table 5:** Coping strategies used by patients’ households for the financial constraints of cancer care in Addis Ababa, Ethiopia 2018

Coping strategies	N	% (95% CI)
Savings^a^	301	85.5 (81.4–88.8)
Financial support^b^	151	43.0 (38.0–48.1)
Selling assets ^c^	42	12.0 (8.9–15.8)
Borrowings^d^	30	8.5 (6.0–12.0)

^a^: It includes any method of household savings and sources from the household member (including *Eqqub* and *Iddir*)

^b^: Financial source or support received from relatives, non-governmental organizations, religious organizations and others source of payments which are nonrefundable

^c^: Any means of payment made by selling household assets like land, property, livestock, jewellery and other household items

^d^: Borrowings took from financial institutions and individuals

NB: Frequencies and percentage would not be added up because multiple responses were possible

## Discussion

This study is the first to assess the incidence of CHE, associated factors, and coping strategies for cancer care in Ethiopia. The average overall cancer care expenditure was half of the average unadjusted household annual income and nearly three-fourth of the average unadjusted household yearly spending, which is higher than that of patients in Australia [25]. However, it was lower than the findings of other studies in which patients spent 59.9% of annual household income [19]. The overall expenditure was very high compared to the per capital income of the population of Ethiopia [26], indicating that uncountable numbers of patients are at home and are dying without getting any treatment and diagnostic services because of the catastrophic expenditure.

Similar to a study reported by Pourreza et al [27], the present research showed that the payment for treatment of colorectal cancer was higher than other types of cancer. The reason being the extended duration of therapy and multiple combination regimens is required to treat colorectal cancer patients, which could result in higher OOP expenditure [28].

Our study found that the level of CHE was very high compared to what was reported from cancer patients in Korea (39.8%) [29]. Besides, although direct comparison seems infeasible because of the different threshold level of CHE used, our finding was higher as compared to other studies reported from Asian countries (47.8–67.9%) [30–32]. But, this was lower compared to patients with breast cancer in India (84%) [33]. This high level of CHE might be due to the limited number of treatment and diagnostic centers available, which can increase its side expenditures and frequent stock out of prescribed medicines. A higher level of CHE was reported due to cancer care when compared to care for cardiovascular patients in Ethiopia (27.0%) [20].

Similarly, with the threshold set at 10% household consumption expenditure, the level of CHE documented in the present study was higher compared to a study conducted in households of persons with depression and high disability, and severe mental disorder [34, 35]. Limited geographic access to cancer care centers and higher cost of medicines might be the reasons for such difference. The government provide oncology medicines in a 50 % subsidized price, but there is still an imbalance in the demand and supply of drugs [36]. As a result, patients have no choice but to buy from the private medicine retail outlets, which might result in patients’ households to face another intolerable financial burden.

The level of CHE also showed variation among income quintiles, although it was not statistically significant. Other studies revealed that the lowest income category was highly associated with catastrophic expenditure [32, 37, 38]. However, in the present study, the fourth income strata faced with a higher level of CHE than the others. Patients with lower incomes might not visit and purchase expensive services if the services aren’t available at affordable price. However, when the disease condition gets complicated these fourth income quintiles might pay while facing catastrophic expenditure.

The level of CHE and self-reported financial pressure was simultaneously high and approximately parallel. This self-reported unmanageable rate of financial burden documented in our study was lower than that recorded in China (75%) [19]. However, it was higher than the study finding in the United States, which showed a financial distress level of 47% [39]. The presence of financial sharing policy and the difference in the income status of the population would influence the level of financial distress across countries, indicating the need to institute risk pooling and sharing mechanisms.

The patients’ age and increased the number of cycles of chemotherapy was among the predicting factors of CHE. Other studies showed similar finding [16, 25]. Increasing CHE with age could be attributed to disease complication and income sources. The numbers of cycles of chemotherapy is also an indicator of the stage of the disease. The possible treatment failure and the need for either re-treatment or new treatments would force the patients to incur additional expenses, thereby increasing the financial burden [40–42]. Although not statistically significant, patients with a history of visit at a private health facility, patients with colorectal cancer, patients attending a private health facility, male, single, and patients residing out of Addis Ababa reportedly encountered with higher-level cancer care expenditure.

Similar to other studies, patient’ households use more than one coping strategies to overcome their financial distress, with the most prevalent being borrowing, selling assets, spending savings, and financial aid [43]. Among those, household saving was the primary coping strategy used in the study, which was also documented in other studies [7, 20, 44]. However, some other studies indicated that borrowing and selling assets were the major coping strategies [27, 44–46]. Variations in economic status and saving culture across households can affect the coping mechanism of patients’ families [47, 48].

Although the study comes with those results it was not without limitations. First, due to the nature of data collection technique, recall bias could be a problem. Obtaining reliable information on the household annual income and expenditure was also another challenge. Since the study design was hospital-based, a significant number of populations may not have access because of affordability and limited availability. Hence, most patients’ households included in the study were more likely to have a relatively higher income.

## Conclusion

Based on a 10% threshold of annual household income, a substantial number of patients with cancer are exposed to a catastrophic level of healthcare expenditure with considerable medical cost. Predominantly, patients with increased cycles of chemotherapy and older age were incurring a catastrophic level of healthcare expenditure. Household saving was the primary coping strategy; however, a significant number of patients’ households had been forced to look for other coping mechanisms. The efficient mobilization of the health insurance scheme is urgently needed to ensure financial risk protection and realize universal health coverage for patients with cancer. Increased tax funding and/or other better prepayment mechanisms should also be considered while mobilizing the already introduced health insurance scheme.

## Supplementary information

**Additional file 1.** Table S1 Data collection instrument

## Data Availability

The dataset should be accessible from the corresponding author on a reasonable request.

## Abbreviations

AOR: Adjusted Odds Ratio
CHE: Catastrophic Health Expenditure
COR: Crude Odds Ratio
Inter-Quartile Range: IQR
OOP: Out of Pocket
SD: Standard Deviation
TASH: Tikur Anbessa Specialized Hospital
WHO: World Health Organization
