# Supplementary material for: Catastrophic healthcare expenditure and coping strategies among patients attending cancer treatment services in Addis Ababa, Ethiopia
Source: BMC Public Health. 2020 Jun 22;20:984. doi: 10.1186/s12889-020-09137-y (PMC7310089; doi:10.1186/s12889-020-09137-y)
Supplement: Supplementary file 1 — Additional file 1. Table S1 Data collection instrument [file 12889_2020_9137_MOESM1_ESM.docx]

**Addis Ababa University**

**College of Health Science**

**School Pharmacy**

**Department of Pharmaceutics and Social Pharmacy**

**Participant information and consent form**

Hello, Dear participant! My name is ____________________. I am representing the School of Pharmacy, Addis Ababa University. I am here to collect data for a research entitled “**Catastrophic Healthcare Expenditure and Coping Strategies among Patients Attending Cancer Treatment Services in Addis Ababa, Ethiopia**”. The research investigator is Gebremicheal Gebreslassie**,** a Masters student in Pharmaco-Epidemiology and Social Pharmacy Program, School of Pharmacy, Addis Ababa University. It is a hospital-based cross-sectional study to be conducted at public and private hospitals providing cancer treatment services. The purpose of the study is to determine the incidence of catastrophic health expenditure, identify associated factors and coping strategies among patients attending cancer treatment services in Addis Ababa, Ethiopia.

Becoming part of study will not have any reward you gain but the study finding will give an insight for policy makers that might have an influence on the current healthcare practice. It might take you 25-30 minutes to finish the interview. You are being part of the study by chance and you will not get any harm because of participating in the study. Your participation in this study is completely voluntary. You have the right to withdraw from the study in any time. We reassure all your responses will remain strictly confidential and will be handled in secured manner. The information you provide will be used only for the purpose of the study stated above. Therefore, I would like to confirm your consent to be part of the study.

Do you agree to be part of the study?

**Agree Disagree**

Thank you very much to be part of the study.

If you have any concerns you can contact the following individuals;

- Research investigator
  - - Name: Mr. Gebremicheal Gebreslassie

Phone number: +251 909 270 062/+251 914 586 170

E-mail: [gebremicheal.kassahun@gmail.com](mailto:gebremicheal.kassahun@gmail.com)

- Research Advisors
  - - Name: Dr. Teferi Gedif

E-mail: [tgedif@gmail.com](mailto:tgedif@gmail.com)

- - - Name: Mr. Gebremedhin Beedemariam

E-mail: [gebremedhin.beedemariam@aau.edu.et](mailto:gebremedhin.beedemariam@aau.edu.et)

- - - Name: Mr. Yohannes Hailemichael

E-mail: [yohannes.h.michael@gmail.com](mailto:yohannes.h.michael@gmail.com)

- - - Name: Dr. Aynalem Abraha

Email: [aynalemab@yahoo.co.uk](mailto:aynalemab@yahoo.co.uk)

**Data collection instrument**

**Title: Catastrophic Healthcare Expenditure and Coping Strategies among Patients Attending Cancer Treatment Services in Addis Ababa, Ethiopia 2018**

| Name of the health facility: ____________  Type of health Facility: Public Private  Interviewer’s name: ____________________  Interviewer’s signature: ____________________ | | | | | | | | | Code no: __________________  Date of the interview: ____/___/___  Interview time began: _______  Interview time taken: _______Minutes |
| --- | --- | --- | --- | --- | --- | --- | --- | --- | --- |
|  | | | | | | | | |  |
| **I: Socio-demographic characteristics** | | | | | | | | | |
| **S.no** | **Questions** | | | **Respondent response** | | | | | |
| 100 | How old are you? | | | _______years/__________E.C | | | | | |
| 101 | What is your gender? | | | _1_Male _2_Female | | | | | |
| 102 | What is your ethnicity? | | | _1_Oromia _2_Amhara _3_Tigray _4_Gurage  _5_Others (specify) __________ | | | | | |
| 103 | What is your religion? | | | _1_Orthodox _2_Muslim _3_Protestant _4_Catholic _5_Others (specify) __________ | | | | | |
| 104 | What is your marital status? | | | _1_Single _2_Married _3_Divorced _4_Widowed | | | | | |
| 105 | Where is your residence area? | | | _1_Addis Ababa _2_Out of Addis Ababa | | | | | |
| 106 | What is your level of education? | | | _1_No formal education _2_1-8 Grade  _3_9-12 Grade _4_College and above | | | | | |
| 107 | What is your current occupation? | | | _1_Farmer _2_Government employee  _3_Private employee _4_Own private business  _5_Retired _6_Housewife/Husband  _7_Student _8_Others (specify)_______ | | | | | |
| 108 | What is the household composition? | | | Total household size _______  _1_Children (<16years old) _______  _2_Adult (17-64 years old)_______  _3_Geratrics (>65years old) _______ | | | | | |
| **II: Medical information** | | | | | | | | | |
| 200 | Which type of cancer was you diagnosed? | | | | _1_Breast cancer _2_Cervical cancer  _3_Colorectal Cancer _4_Prostate cancer  _5_Others (please specify) ___________ | | | | |
| 201 | When was your case confirmed? | | | | ___/____/___ E.C or ______months before | | | | |
| 202 | What was the stage of your disease? | | | | ________ stage | | | | |
| 203 | When did the treatment protocol initiated? | | | | _______ months/_________days before  _2_On the day disease confirmed | | | | |
| 204 | Which did you receive so far?  (Multiple answers possible) | | | | _1_Chemotherapy _2_Radiotherapy  _3_Surgery _4_Hormonal  _5_Supportive treatment  _6_Others (specify)_________ | | | | |
| 205 | On which treatment cycles are you now/ were you? | | | | _________cycle (for chemotherapy)  _2_On other treatment options | | | | |
| 206 | Did you ever visit private health facilities before? | | | | _1_Yes _2_No | | | | |
| **III: Outpatient department (OPD) care expenditure (skip to part IV if you don’t incur for OPD service before)** | | | | | | | | | |
| 300 | Over the last 12 months (total visit), how many times did you visit oncology service providing health facilities for your cancer? | | | | | ______times | | | |
| 301 | During this month of outpatient visit, how much ETB did you spent for the following services?  (Please write your answer in ETB) | | | | | **Total___________**  _1_Consultation cost_______  _2_Investigation/imaging cost_____  _3_Medicines cost________  _4_Transportation cost_______  _5_Patient income lost________  _6_Care giver income lost______  _7_Food & other related costs _________ | | | |
| 302 | How much was the total OPD expenditure for your cancer in the last 12 months? | | | | | ___________ETB/12 months | | | |
| 303 | During the last 12 months, how much did you spend for traditional treatment? | | | | | ____________ETB/12 months  (If no, skip to part IV) | | | |
| **IV: Inpatient Department (IPD) cancer care expenditure (skip to part V if you don’t incur for IPD service before)** | | | | | | | | | |
|  | | | | | | | | | |
| 400 | Over the last 12 months, how many times did you visit oncology service providing health facilities for your cancer? | | | | | ______times | | | |
| 401 | During this month inpatient admission, how much ETB did you spent for the following services:  (Please write your answer in ETB) | | | | | **Total__________**  _1_Consultation cost_______  _2_Investigation/imaging cost_____  _3_Medicines cost________  _4_Transportation cost_______  _5_Hospital bed cost ________  _6_Patient income lost_____  _7_Care giver income lost______  _8_Food & other related costs_________ | | | |
| 402 | How much was the total inpatient admission cost (12 months cost)? | | | | | ________ETB/12 months | | | |
| 403 | During the last 12 months, how much did you spend for traditional treatment? | | | | | ____________ETB/12 months  (If no, skip to part V) | | | |
| **V: Patients’ household essential consumptions and income (Please put your answer in ETB)** | | | | | | | | | |
| 500 | On average in the last 7 days, how much does your household spend for food and food items; (staple foods, fruits and vegetables...) | | | | | | | ____________ETB/Week | |
| 501 | In the last 30 days, what was household expense for: | | Total______________/Month  _1_Housing and utilities (Rent, electricity, heating, water, telephone…) ____________  _2_Clothing ____________  _3_Transportation ___________  _4_Household health care cost__________  _5_Recreation and entertainment ___________  _6_Other goods and services? (specify) ___________ | | | | | | |
| 502 | In the last 12 months, how much did your household spend for the following? | | _1_Education fees and supplies ___________  _2_Durable goods (televisions, phones, furniture, vehicles...) __________  _3_Rituals, gifts or ceremonies (funerals, birthdays, wedding) _________  _4_Health-related items _________  _5_Other goods and services (property, land, livestock, cleaning services, repair services…) ________ | | | | | | |
| 503 | Overall, what was your household's overall expenditure? | | | | | | ___________ETB/1month  ___________ETB/12 months | | |
| 504 | How much is the patient’s current monthly income? | | | | | | ___________ETB/month  (if not, proceed to Question number, 505) | | |
| 505 | How much is the household total monthly income? | | | | | | ___________ETB/1 month  ___________ETB/12 months | | |
| **VI: Patients’ Household financial situation outlook** | | | | | | | | | |
| 600 | How would you rate the financial situation of the household compared to the past? | | | | | | _1_Very good _2_Good _3_Average/similar  _4_Bad _5_Very bad | | |
| 602 | Because of the disease imposed financial difficulty to the household, which measures have you ever taken? (E.g.; you spent 28,000 ETB for cancer treatment)  (Multiple answers possible)  (Please write your answer in ETB) | _1_Current income of any household member/s __________  _2_Savings ___________  _3_Ask relatives, Religious and NGOs __________  _4_Borrow from financial institutions (Banks, microfinance schemes? ___________  _5_Payment or reimbursement from a health insurance plan (including private health schemes)? __________  _6_Cut down on food and other HH consumption ________  _7_Sold items (land, property, livestock, jewellery) ___________  _8_Withdraw Children from school __________  _9_Reduce Medical visits/treatment _________  _10_*Eqqub*/*Iddir* _________  _11_Others (specify) __________ | | | | | | | |

Interview time taken: ___________ Interview time ended: ________

Thank you very much for your time and answers!
